# Hematological biomarkers for predicting pathologic response to neoadjuvant immunochemotherapy and cycle optimization in locally advanced gastric cancer

**DOI:** 10.3389/fimmu.2026.1795481

**Published:** 2026-05-25

**Authors:** Xinglong Lu, Xiang Cui, Hao Chen, Jianling Zhang, Shengbing Zhao, Baoshun Yang, Yang Yang, Nan Du, Wenxiang Ma, Jixi An, Yongjiang Yu

**Affiliations:** 1 The First School of Clinical Medicine, Lanzhou University, Lanzhou, China; 2 Department of General Surgery, The First Hospital of Lanzhou University, Lanzhou, China

**Keywords:** gastric cancer, hematological parameters, neoadjuvant immunochemotherapy, neutrophil, pathologic response

## Abstract

**Background:**

The predictive value of hematological parameters for pathologic response to neoadjuvant immunochemotherapy (NICT) in locally advanced gastric cancer (LAGC) remains unclear.

**Methods:**

This retrospective study consecutively enrolled 246 LAGC patients who received NICT followed by radical gastrectomy at The First Hospital of Lanzhou University (2021-2024). Based on postoperative pathology, patients were classified into major pathologic response (MPR, tumor regression grade [TRG] 1a/1b, residual tumor ≤10%) and incomplete pathologic response (IPR, TRG 2/3, residual tumor >10%) groups. Hematological parameters pre- and post-treatment were analyzed. Logistic regression and receiver operating characteristic (ROC) analyses were employed (statistical significance: p<0.05).

**Results:**

Multivariate analysis identified pre-treatment neutrophil count (Neutrophil_pre) (adjusted odds ratio [OR]: 0.83, 95% CI: 0.70–0.99, P = 0.033) and post-treatment albumin (ALB_post) (adjusted OR: 0.92, 95% CI: 0.85–1.00, P = 0.042) as independent protective factors for MPR, while post-treatment platelet count (PLT_post) was an independent risk factor (adjusted OR: 1.01, 95% CI: 1.00–1.01, P = 0.014). These associations were absent in a chemotherapy-alone control cohort (n=147). ROC analysis determined the optimal pre-treatment neutrophil cutoff at 3.39×10^9^/L. Subgroup analysis showed that among patients with pre-treatment neutrophils <3.39×10^9^/L, those receiving 4 (vs. 2-3) cycles of neoadjuvant therapy had significantly higher MPR (35.7% vs. 16.9%, P = 0.039) and pathologic complete response (pCR) rates (32.1% vs. 9.1%, P = 0.011).

**Conclusion:**

Pre-treatment neutrophil count, post-treatment platelet count, and post-treatment albumin level are independent predictors of pathologic response to NICT in LAGC. Patients with low pre-treatment neutrophil levels may benefit from extended (4-cycle) neoadjuvant immunochemotherapy with higher pathologic response rates.

## 1 Introduction

Gastric cancer is one of the most common malignancies worldwide, with high incidence and mortality rates (1). The treatment strategy for locally advanced gastric cancer (LAGC) has evolved from surgery alone to a comprehensive treatment model centered on surgery. The application of neoadjuvant chemotherapy has significantly improved surgical resection rates and the degree of pathologic response, with potential to enhance patient survival (2, 3). In recent years, with the breakthroughs of immune checkpoint inhibitors in the treatment of advanced gastric cancer, neoadjuvant immunochemotherapy (NICT) has gradually become a highly promising strategy for LAGC. Studies have shown that compared to traditional neoadjuvant chemotherapy, NICT can further increase pathologic response, offering better survival benefits for patients (4–7). However, not all patients benefit equally from NICT, with a considerable proportion showing poor or no response. Identifying potential responders and non-responders early in treatment is a key issue in clinical practice (8–10).

Peripheral blood routine parameters, as important indicators reflecting the body’s systemic inflammatory and immune status, have the advantages of being simple, economical, and amenable to dynamic monitoring. Previous studies suggest that systemic inflammatory markers (such as neutrophils, platelets, and derived indices) have certain value in predicting prognosis in various solid tumors treated with neoadjuvant immunochemotherapy and in gastric cancer treated with neoadjuvant chemotherapy. However, these studies mostly focused on combined analyses of multiple indicators, while research on the independent role of single hematological parameters and their predictive value for the efficacy of NICT in gastric cancer is still insufficient (11, 12).

Notably, the differential responses to neoadjuvant immunochemotherapy among patients may stem from heterogeneity in the baseline tumor microenvironment (TME) (13). The composition, activation status, and spatial distribution of immune cells in the TME of gastric cancer exhibit substantial inter-individual variability, which directly influences the ultimate efficacy of immune checkpoint inhibitors. Accumulating evidence indicates that immune cells within the TME (e.g., neutrophils, macrophages, lymphocytes) possess a high degree of duality: they can either promote tumor progression or mediate effective anti-tumor immune responses, with the net effect highly dependent on the microenvironmental context (14). For instance, specific subsets of neutrophils have been shown to be closely associated with immunotherapy response (15). Moreover, the mechanism of action of neoadjuvant immunochemotherapy (NICT) fundamentally differs from that of chemotherapy alone: the former primarily reshapes the TME and activates systemic immune responses, whereas the latter mainly relies on direct cytotoxic effects on tumor cells (16). Peripheral blood inflammatory parameters (e.g., neutrophils, platelets, albumin) can partially reflect the immune status of the TME, and previous studies have suggested their potential predictive value in immunotherapy (17). Therefore, exploring the association between individual peripheral blood parameters and pathologic response in the context of NICT has important clinical value and research necessity.

Additionally, controversy remains regarding the optimal number of NICT cycles. A review based on international consensus and clinical studies recommends 2–4 cycles of neoadjuvant therapy, but differences between cycle numbers were not mentioned (18). Some studies have confirmed that in certain subgroups or cancer types, extending the treatment cycle can improve the level of pathologic response (19–21). Therefore, the impact of different cycle numbers on neoadjuvant treatment efficacy requires further study.

In summary, this study aims to retrospectively analyze the clinical data of LAGC patients receiving NICT, evaluate the predictive value of hematological markers for pathologic response to NICT in gastric cancer, and preliminarily explore the impact of treatment cycle number on efficacy.

## 2 Materials and methods

### 2.1 Study design and patient population

This was a single-center retrospective study. By querying the electronic medical record database, 246 patients who received NICT between January 1, 2021, and December 31, 2024, and 147 patients who received neoadjuvant chemotherapy alone between January 1, 2018, and December 31, 2022, were consecutively enrolled.

Inclusion criteria: (1) Pathologically confirmed locally advanced gastric adenocarcinoma (22); (2) Clinical stage Ib, II, or III according to the American Joint Committee on Cancer (AJCC) 8th edition staging system, with no evidence of distant metastasis on imaging (23); (3) Eastern Cooperative Oncology Group (ECOG) performance status score of 0-1 (24); (4) Successful completion of 2–4 cycles of neoadjuvant chemotherapy: SOX regimen (oxaliplatin + tegafur/gimeracil/oteracil), CAPOX regimen (oxaliplatin + capecitabine), or other regimens, with or without PD-1 checkpoint inhibitor therapy (sintilimab, tislelizumab, or other PD-1 inhibitors), followed by radical gastrectomy; (5) Complete availability of laboratory test data from within 1 week before treatment and 1 week before surgery.

Exclusion criteria: (1) Received any form of immunotherapy or other anti-tumor treatment before starting neoadjuvant therapy; (2) History of immunodeficiency diseases, active autoimmune diseases, or receiving treatment for autoimmune diseases, or requiring systemic immunosuppressive therapy within 7 days before starting neoadjuvant therapy; (3) Severe hepatic or renal insufficiency or organic damage to other vital organs; (4) History of other malignancies within the past five years; (5) Presence of active infectious diseases (e.g., HIV, active hepatitis B, etc.); (6) Active hematological diseases, active inflammation, or infectious conditions that may significantly interfere with the interpretation of blood parameters.

This study was reviewed and approved by the Ethics Committee of The First Hospital of Lanzhou University (Approval No.: LDYYLL2026-02). The requirement for informed consent was waived due to the retrospective nature of the study.

### 2.2 Data collection

The following data were collected from medical records: age, gender, baseline height, baseline weight, tumor location, neoadjuvant treatment regimen, number of neoadjuvant treatment cycles, and pathologic response. Hematological parameters from fasting peripheral venous blood samples taken before neoadjuvant therapy and after neoadjuvant therapy (pre-surgery) were extracted, including: total white blood cell count (WBC), absolute neutrophil count (Neutrophil), absolute lymphocyte count (LYM), absolute monocyte count (MON), hemoglobin concentration (Hb), platelet count (PLT), and albumin (ALB). The specific peripheral blood inflammatory index was calculated as: NLR = N/L (neutrophil count/lymphocyte count) (25).

### 2.3 Pathologic response assessment

Pathologic response was assessed according to the Becker tumor regression grading criteria. Patients were divided into two groups for primary analysis: the major pathologic response group (MPR, TRG1a and TRG1b, residual viable tumor cells ≤10%) and the incomplete pathologic response group (IPR, TRG2 and TRG3, residual viable tumor cells >10%). Pathologic complete response (pCR) was defined as the absence of residual viable tumor cells in both the resected primary tumor and all sampled lymph nodes (26).

### 2.4 Statistical analysis

Categorical variables are expressed as frequencies and percentages and were analyzed using the chi-square test or Fisher’s exact test. Continuous variables with skewed distribution were analyzed using the Mann-Whitney U test. Receiver operating characteristic (ROC) curves were plotted to determine the optimal cutoff values for each indicator. Univariate and multivariate logistic regression analyses were employed to identify independent predictors of pathologic response for NICT and neoadjuvant chemotherapy alone. Multicollinearity was assessed using the variance inflation factor (VIF), with VIF >10 indicating severe collinearity. Variables causing collinearity were excluded. The remaining candidate variables were included in a multivariate logistic regression model (significance level α=0.05) to identify independent predictors. A P-value less than 0.05 was considered statistically significant. IBM SPSS Statistics 27 and R 4.5.1 were used for statistical analysis. Graphical figures were generated using GraphPad Prism (version 10.1.2) and Adobe Photoshop (version 2024).

## 3 Results

### 3.1 Baseline characteristics

The main clinical characteristics of the patients are shown in **Table 1**. The NICT group included 246 patients with a mean age of 59.4 ± 8.6 years, including 196 males (79.7%) and 50 females (20.3%). The proportions of tumors located in the upper, middle, and lower stomach were 35.8%, 22.8%, and 37.4%, respectively, with 4.1% in mixed locations. The vast majority of patients (83.7%) received the SOX chemotherapy regimen, 7.7% received CAPOX, and 8.5% received other regimens (detailed in **Supplementary Table 1**). Regarding neoadjuvant treatment cycles, most patients received 3 cycles (63.0%) or 4 cycles (29.7%). Sintilimab was the primary immunotherapy drug, used by 223 patients (90.7%). The chemotherapy-alone group included 147 patients, with 120 males (81.6%) and 27 females (18.4%), and a mean age of 56.9 ± 8.3 years. The SOX regimen was the most commonly used chemotherapy regimen (80.3%), and 76.2% of patients received 3 cycles of neoadjuvant chemotherapy.

**Table 1:** The correlation between pathological response and variables in the two different treatment groups.

Variables	Neoadjuvant chemotherapy combined with immunotherapy group				Neoadjuvant chemotherapy group			
	Total (n=246)	MPR (n=81)	IPR (n=165)	P value	Total (n=147)	MPR (n=35)	IPR (n=112)	P value
**Gender**				0.622				0.775
Male	196 (79.7)	66 (81.5)	130 (78.8)		120 (81.6)	28 (80)	92 (82.1)	
Female	50 (20.3)	15 (18.5)	35 (21.2)		27 (18.4)	7 (20)	20 (17.9)	
**Age**	59.4 ± 8.6	60.2 ± 7.3	59.0 ± 9.2	0.275	56.9 ± 8.3	58.5 ± 7.5	56.4 ± 8.6	0.208
**Tumor location**				0.737				0.227
Upper	88 (35.8)	27 (33.3)	61 (37)		27 (18.4)	6 (17.1)	21 (18.8)	
Middle	56 (22.8)	22 (27.2)	34 (20.6)		70 (47.6)	13 (37.1)	57 (50.9)	
Lower	92 (37.4)	29 (35.8)	63 (38.2)		47 (32.0)	16 (45.7)	31 (27.7)	
Mixed	10 (4.1)	3 (3.7)	7 (4.2)		3 (2.0)	0 (0)	3 (2.7)	
**Chemo regimen**				0.057				0.383
SOX	206 (83.7)	72 (88.9)	134 (81.2)		118 (80.3)	26 (74.3)	92 (82.1)	
CAPOX	19 (7.7)	7 (8.6)	12 (7.3)		13 (8.8)	3 (8.6)	10 (8.9)	
Other	21 (8.5)	2 (2.5)	19 (11.5)		16 (10.9)	6 (17.1)	10 (8.9)	
**Chemo cycles**				0.259				0.296
2	18 (7.3)	3 (3.7)	15 (9.1)		17 (11.6)	2 (5.7)	15 (13.4)	
3	155 (63.0)	51 (63)	104 (63)		112 (76.2)	27 (77.1)	85 (75.9)	
4	73 (29.7)	27 (33.3)	46 (27.9)		18 (12.2)	6 (17.1)	12 (10.7)	
**Immuno regimen**				0.811				
Sintilimab	223 (90.7)	75 (92.6)	148 (89.7)					
Tislelizumab	9 (3.7)	2 (2.5)	7 (4.2)					
Other	14 (5.7)	4 (4.9)	10 (6.1)					
**Immuno cycles**				0.764				
2	23 (9.3)	6 (7.4)	17 (10.3)					
3	155 (63.0)	52 (64.2)	103 (62.4)					
4	68 (27.6)	23 (28.4)	45 (27.3)					
**BMI**	22.2 ± 2.7	22.1 ± 2.5	22.2 ± 2.8	0.794	22.1 ± 3.1	22.0 ± 2.9	22.2 ± 3.1	0.675
**WBC_pre**	5.9 ± 2.0	6.4 ± 2.3	5.6 ± 1.7	**0.004**	6.0 ± 1.8	6.0 ± 1.7	6.0 ± 1.9	0.922
**Hb_pre**	131.5 ± 27.3	136.0 ± 23.7	129.2 ± 28.8	0.069	134.5 ± 30.5	138.1 ± 30.2	133.4 ± 30.6	0.428
**PLT_pre**	245.1 ± 97.0	246.7 ± 95.9	244.3 ± 97.9	0.861	232.6 ± 94.0	237.8 ± 73.2	231.0 ± 99.8	0.712
**LYM_pre**	1.4 ± 0.5	1.5 ± 0.6	1.4 ± 0.5	**0.023**	1.5 ± 0.5	1.7 ± 0.5	1.5 ± 0.5	**0.024**
**MON_pre**	0.3 ± 0.1	0.4 ± 0.2	0.3 ± 0.1	0.116	0.4 ± 0.1	0.4 ± 0.1	0.4 ± 0.1	0.502
**Neutrophil_pre**	4.0 ± 1.7	4.3 ± 1.9	3.8 ± 1.5	**0.014**	4.0 ± 1.6	3.8 ± 1.5	4.0 ± 1.7	0.444
**NLR_pre**	3.0 ± 1.5	3.0 ± 1.3	3.0 ± 1.6	0.857	3.0 ± 1.7	2.5 ± 1.4	3.2 ± 1.8	0.059
**ALB_pre**	42.8 ± 4.0	42.8 ± 3.6	42.9 ± 4.2	0.875	42.8 ± 4.2	42.4 ± 3.4	43.0 ± 4.5	0.528
**WBC_post**	4.8 ± 1.9	4.8 ± 1.6	4.7 ± 2.0	0.709	4.8 ± 1.8	5.1 ± 2.1	4.7 ± 1.7	0.316
**Hb_post**	123.2 ± 21.8	127.3 ± 19.6	121.1 ± 22.6	**0.036**	126.6 ± 23.7	128.8 ± 21.9	125.8 ± 24.3	0.518
**PLT_pos**	144.7 ± 75.2	128.8 ± 66.4	152.4 ± 78.1	**0.020**	137.7 ± 64.7	144.4 ± 77.9	135.7 ± 60.2	0.489
**LYM_post**	1.3 ± 0.5	1.4 ± 0.5	1.3 ± 0.5	0.276	1.4 ± 0.5	1.5 ± 0.5	1.4 ± 0.5	0.368
**MON_pos**	0.4 ± 0.2	0.4 ± 0.2	0.4 ± 0.1	0.465	0.4 ± 0.2	0.5 ± 0.3	0.4 ± 0.1	**0.009**
**Neutrophil_post**	2.9 ± 1.7	2.9 ± 1.3	2.9 ± 1.8	0.889	2.8 ± 1.7	2.9 ± 1.9	2.7 ± 1.7	0.676
**NLR_post**	2.4 ± 1.5	2.4 ± 1.3	2.5 ± 1.6	0.643	1.7 (1.2, 2.4)	1.6 (1.2, 2.3)	1.7 (1.2, 2.4)	0.639
**ALB_post**	41.3 ± 4.0	42.1 ± 3.5	40.8 ± 4.2	**0.023**	41.8 ± 3.8	41.2 ± 3.1	42.0 ± 4.0	0.283

Bold values indicate statistical significance (P < 0.05).

Further comparison of MPR and IPR patients within each group revealed significant differences in pre-treatment hematological indicators in the NICT group: Compared to the IPR group, the MPR group had significantly higher pre-treatment white blood cell count (6.4 ± 2.3 vs. 5.6 ± 1.7 ×10^9^/L, P = 0.004), neutrophil count (4.3 ± 1.9 vs. 3.8 ± 1.5 ×10^9^/L, P = 0.014), and lymphocyte count (1.5 ± 0.6 vs. 1.4 ± 0.5 ×10^9^/L, P = 0.023). In the chemotherapy-alone group, the MPR subgroup also showed a higher lymphocyte count (1.7 ± 0.5 vs. 1.5 ± 0.5 ×10^9^/L, P = 0.024), but no significant differences were found between the MPR and IPR groups regarding white blood cell or neutrophil counts.

### 3.2 Univariate and multivariate regression analysis

In the NICT group, univariate analysis (α = 0.10) showed that WBC_pre, Hb_pre, LYM_pre, Neutrophil_pre, Hb_post, PLT_post, and ALB_post were associated with MPR. Before constructing the multivariate logistic regression model, collinearity diagnostics revealed severe multicollinearity among WBC_pre, Neutrophil_pre, and LYM_pre (variance inflation factor [VIF] values were 134.56, 105.40, and 13.38, respectively). To eliminate collinearity, WBC_pre, which had the most significant information overlap, was removed. The VIF values of the remaining variables were all reduced to below 1.09, meeting the modeling requirements. Multivariate analysis (α = 0.05) results showed that PLT_post was an independent risk factor for MPR (adjusted OR = 1.01, 95% CI: 1.00–1.01, P = 0.014). Neutrophil_pre was an independent protective factor for MPR (adjusted OR = 0.83, 95% CI: 0.70–0.99, P = 0.033). ALB_post was an independent protective factor for MPR (adjusted OR = 0.92, 95% CI: 0.85–1.00, P = 0.042).

**Figure 1:**
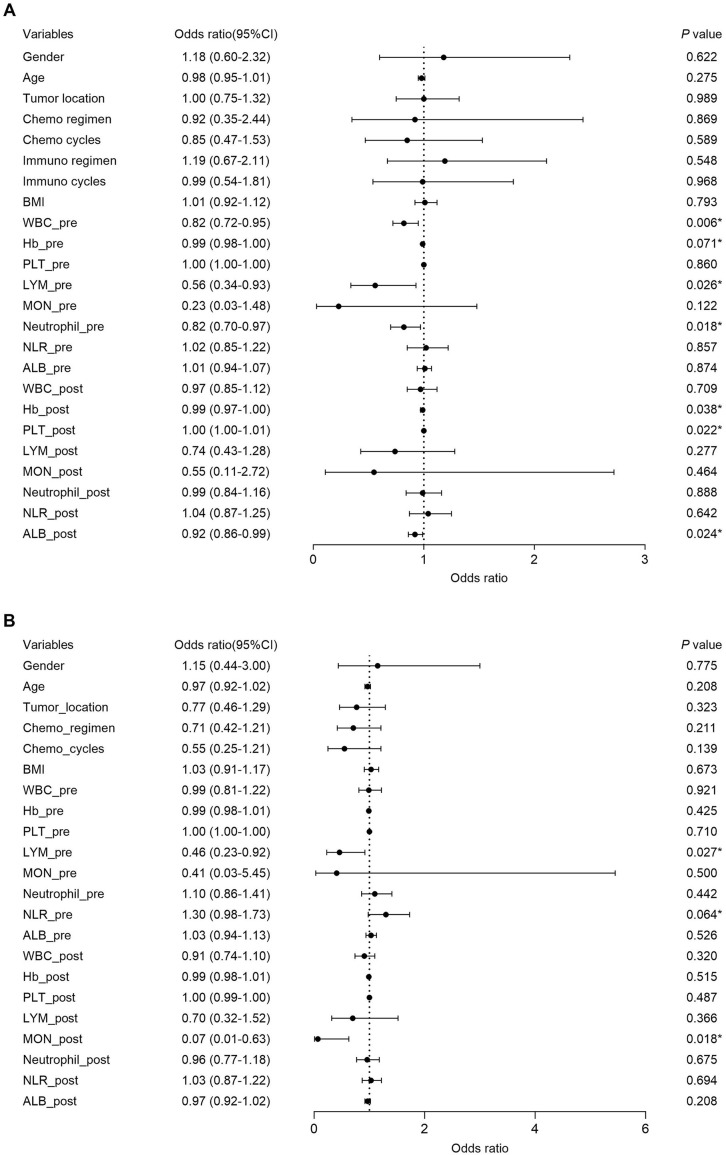
Clinical factors associated with major pathologic response (MPR) using univariate logistic regression models. **(A)** Univariate analysis in the neoadjuvant chemotherapy combined with immunotherapy group. **(B)** Univariate analysis in the neoadjuvant chemotherapy group.

Univariate analysis (α=0.10) in the chemotherapy-only group identified LYM_pre, NLR_pre, and MON_post as factors associated with MPR. Collinearity diagnostics indicated that all variables had VIF values below 5, suggesting no significant multicollinearity. Multivariate analysis (α=0.05) ultimately confirmed MON_post as an independent protective factor for MPR (adjusted OR = 0.10, 95% CI: 0.01–0.90, P = 0.040). Univariate and multivariate logistic regression analyses are shown in **Table 2**; **Figure 1**.

**Table 2:** Univariate and multivariate analysis of MPR for neoadjuvant chemotherapy or combined with immunotherapy group.

Variables	Univariate and multivariate for MPR in neoadjuvant chemotherapy combined with immunotherapy group				Univariate and multivariate for MPR in neoadjuvant chemotherapy group			
	Odds ratio (95%CI)	P value (α=0.10)	Odds ratio (95%CI)	P value (α=0.05)	Odds ratio (95%CI)	P value (α=0.10)	Odds ratio (95%CI)	P value (α=0.05)
Gender	1.18 (0.6-2.32)	0.622			1.15 (0.44-3)	0.775		
Age	0.98 (0.95-1.01)	0.275			0.97 (0.92-1.02)	0.208		
Tumor location	1.00 (0.75 - 1.32)	0.989			0.77 (0.46-1.29)	0.323		
Chemo regimen	0.92 (0.35-2.44)	0.869			0.71 (0.42-1.21)	0.211		
Chemo cycles	0.85 (0.47-1.53)	0.589			0.55 (0.25-1.21)	0.139		
Immuno regimen	1.19 (0.67 - 2.11)	0.548						
Immuno cycles	0.99 (0.54-1.81)	0.968						
BMI	1.01 (0.92-1.12)	0.793			1.03 (0.91~1.17)	0.673		
WBC_pre	0.82 (0.72-0.95)	**0.006**			0.99 (0.81~1.22)	0.921		
Hb_pre	0.99 (0.98-1)	**0.071**	1.00 (0.98-1.02)	0.839	0.99 (0.98~1.01)	0.425		
PLT_pre	1 (1-1)	0.860			1 (1-1)	0.710		
LYM_pre	0.56 (0.34-0.93)	**0.026**	0.68 (0.39-1.18)	0.172	0.46 (0.23-0.92)	**0.027**	0.75 (0.31-1.82)	0.522
MON_pre	0.23 (0.03-1.48)	0.122			0.41 (0.03~5.45)	0.500		
Neutrophil_pre	0.82 (0.7-0.97)	**0.018**	0.83 (0.70-0.99)	**0.033**	1.1 (0.86~1.41)	0.442		
NLR_pre	1.02 (0.85-1.22)	0.857			1.3 (0.98-1.73)	**0.064**	1.21 (0.87-1.70)	0.263
ALB_pre	1.01 (0.94-1.07)	0.874			1.03 (0.94~1.13)	0.526		
WBC_post	0.97 (0.85-1.12)	0.709			0.91 (0.74~1.1)	0.320		
Hb_post	0.99 (0.97-1)	**0.038**	1.00 (0.98-1.02)	0.928	0.99 (0.98~1.01)	0.515		
PLT_post	1 (1-1.01)	**0.022**	1.01 (1.00-1.01)	**0.014**	1 (0.99~1)	0.487		
LYM_post	0.74 (0.43-1.28)	0.277			0.7 (0.32~1.52)	0.366		
MON_post	0.55 (0.11-2.72)	0.464			0.07 (0.01~0.63)	**0.018**	0.10 (0.01-0.90)	**0.040**
Neutrophil_post	0.99 (0.84-1.16)	0.888			0.96 (0.77-1.18)	0.675		
NLR_post	1.04 (0.87-1.25)	0.642			1.03 (0.87-1.22)	0.694		
ALB_post	0.92 (0.86-0.99)	**0.024**	0.92 (0.85-1.00)	**0.042**	0.97 (0.92-1.02)	0.208		

Bold values indicate statistical significance at the corresponding α level: P < 0.10 in univariate analysis and P < 0.05 in multivariate analysis.

### 3.3 Predictive value of blood indicators for treatment cycle decision-making

Considering heterogeneous patient responses to treatment, we further investigated the impact of the number of treatment cycles on efficacy in identified key subgroups. Receiver operating characteristic (ROC) curve analysis was used to determine the optimal cutoff values for Neutrophil_pre and PLT_post in predicting pathologic response. The analysis showed that the area under the curve (AUC) for Neutrophil_pre was 0.603 (95% CI: 0.528–0.679, P = 0.008), with an optimal cutoff value of <3.39 × 10^9^/L determined by maximizing the Youden index. The AUC for PLT_post was 0.591 (95% CI: 0.518–0.665, P = 0.020), with an optimal cutoff value of ≥143 × 10^9^/L. Additionally, the AUC for ALB_post was 0.593 (95% CI: 0.518–0.667, P = 0.018), with an optimal cutoff value of ≥39.65 g/L. The detailed ROC curve analysis is presented in **Supplementary Figure 1**. Accordingly, subgroups were divided, and pathologic response rates and pCR rates were compared between patients receiving 2–3 cycles versus 4 cycles of neoadjuvant treatment within each subgroup.

The results showed that in the total population, there were no statistically significant differences in MPR rate (32.6% vs. 33.8%, P = 0.853) or pCR rate (20.2% vs. 29.4%, P = 0.124) between patients receiving 2–3 cycles and 4 cycles of neoadjuvant therapy. In the low pre-treatment neutrophil group, patients receiving 4 cycles had higher MPR and pCR rates (35.7% and 32.1%) compared to those receiving 2–3 cycles (16.9% and 9.1%) (P = 0.039 and P = 0.011, respectively). In other subgroups, no statistically significant differences in MPR or pCR rates were observed between different treatment cycle numbers (all P > 0.05). The association between treatment cycles and pathological response in subgroups is presented in **Table 3**.

**Table 3:** Relationship between treatment cycles and pathological response rates within subgroups.

Groups	Total, n/N (%)	2–3 cycles, n/N (%)	4 cycles, n/N (%)	P value
Total				
MPR	81/246 (32.9)	58/178 (32.6)	23/68 (33.8)	0.853
pCR	56/246 (22.8)	36/178 (20.2)	20/68 (29.4)	0.124
Low Neutrophil_pre				
MPR	23/105 (21.9)	13/77 (16.9)	10/28 (35.7)	**0.039**
pCR	16/105 (15.2)	7/77 (9.1)	9/28 (32.1)	**0.011**
High Neutrophil_pre				
MPR	58/141 (41.1)	45/101 (44.6)	13/40 (32.5)	0.190
pCR	40/141 (28.4)	29/101 (28.7)	11/40 (27.5)	0.885
Low PLT_post				
MPR	55/138 (39.9)	39/99 (39.4)	16/39 (41)	0.860
pCR	38/138 (27.5)	23/99 (23.2)	15/39 (38.5)	0.071
High PLT_post				
MPR	26/108 (24.1)	19/79 (24.1)	7/29 (24.1)	0.992
pCR	18/108 (16.7)	13/79 (16.5)	5/29 (17.2)	1.000
Low ALB_post				
MPR	16/75 (21.3)	12/52 (23.1)	4/23 (17.4)	0.762
pCR	11/75 (14.7)	7/52 (13.5)	4/23 (17.4)	0.728
High ALB_post				
MPR	65/171 (38.0)	46/126 (36.5)	19/45 (42.2)	0.498
pCR	45/171 (26.3)	29/126 (23)	16/45 (35.6)	0.101

Bold values indicate statistical significance (P < 0.05).

## 4 Discussion

Currently, gastric cancer remains a major global health challenge (1). Compared to neoadjuvant chemotherapy alone, NICT improves pathologic response rates and offers promising survival benefits (4–7). However, despite this progress, a significant proportion of patients show no response to this intensified treatment regimen. This retrospective study analyzed the clinical data of LAGC patients receiving NICT versus chemotherapy alone, aiming to explore the predictive value of peripheral blood markers for pathologic response. The results showed that in the NICT group, PLT_post was an independent risk factor for MPR, while Neutrophil_pre and ALB_post were independent protective factors for MPR; these associations were not observed in the chemotherapy-alone group. Subgroup analysis found that for patients with low pre-treatment neutrophil levels, receiving 4 cycles of NICT significantly increased both MPR and pathologic complete response (pCR) rates. These associations were not found in the chemotherapy-alone cohort, indicating that the predictive value of these indicators is specific to the immunochemotherapy cohort.

The findings of this study indicate that a higher pre-treatment neutrophil count is an independent protective factor for achieving major pathologic response (MPR) with NICT. This finding appears to contradict previous studies that associated high neutrophil counts with systemic inflammation and poor prognosis in various cancers (27, 28). However, those studies were largely conducted in patients receiving chemotherapy or surgery alone, where neutrophils primarily reflect a pro-inflammatory, tumor-promoting environment. In the context of NICT, the role of neutrophils is fundamentally different due to the engagement of the immune system by immune checkpoint inhibitors (ICIs), which reinvigorate antitumor immunity by targeting T-cell inhibitory pathways (29), though primary and acquired resistance mechanisms may limit their efficacy (30).

Currently, there are few clinical studies focusing on single hematological indicators like neutrophils. Our finding that higher pre-treatment neutrophils predict better response to NICT is supported by emerging mechanistic evidence. First, neutrophils exhibit remarkable heterogeneity and plasticity in the tumor microenvironment. Recent single-cell studies have revealed that neutrophils can be reprogrammed between pro-tumor N2-like and anti-tumor N1-like phenotypes depending on context (14, 31–33). Specifically, a subset of interferon-stimulated neutrophils has been found to enhance antigen presentation and T-cell priming, thereby creating a more favorable immune environment for ICIs (34, 35). Second, an appropriately elevated neutrophil count may reflect an “immune-active” tumor microenvironment, which is a prerequisite for generating an effective anti-tumor immune response upon PD-1 blockade (36). In contrast, a very low neutrophil state might indicate an immune-cold microenvironment that is less responsive to ICIs.

In summary, in the specific setting of NICT, a higher pre-treatment neutrophil count does not signify adverse prognosis but rather indicates a more robust and activatable immune system. This is consistent with the concept that the predictive value of hematological biomarkers is highly context-dependent.

Notably, while our study focused on absolute neutrophil count, we also examined the neutrophil-to-lymphocyte ratio (NLR). Current research across various cancers shows conflicting roles for NLR, with some studies linking elevated levels to poorer efficacy (27, 28), while others show no association with pathologic response or postoperative complications following neoadjuvant therapy (37, 38). This study did not find an association between NLR and pathologic response, possibly because neutrophil and lymphocyte counts increased synchronously in the MPR group, resulting in a ratio not significantly different from the IPR group. Therefore, analyzing these two single indicators separately may more accurately reflect immune status differences between groups than using their ratio.

Based on the above results and hypothesis, we further investigated the specific subgroup with low pre-treatment neutrophil levels (<3.39×10^9^/L) and found that those receiving 4 cycles had significantly better MPR and pCR rates than those receiving 2–3 cycles (MPR: 35.7% vs. 16.9%, P = 0.039; pCR: 32.1% vs. 9.1%, P = 0.011). While most current studies do not reflect overall differences between cycle numbers across cancer types, some studies have observed that in certain specific subpopulations or cancer types, completing four cycles of neoadjuvant therapy can lead to higher pathologic response rates compared to shorter courses (19, 20, 39). These results suggest that a low pre-treatment neutrophil state may reflect a lower degree of immune environment inflammation or immune priming. These patients may require longer exposure to immunochemotherapy or immune checkpoint inhibitors to fully prime the immune system and recruit effector cells (36, 40–43). In the course of NICT for advanced gastric cancer, an extended treatment duration (four cycles) may provide more time for immune activation in patients with low pre-treatment neutrophils, fostering a more favorable immune environment and leading to greater pathologic regression.

Our study observed that a higher post-treatment platelet count (PLT_post ≥143×10^9^/L) is an independent risk factor for pathologic response. Generally, the role of platelets is mainly associated with thrombosis, tumor cell metastasis, and angiogenesis. An elevated post-treatment platelet count is often viewed as a marker of suboptimal neoadjuvant efficacy (44, 45). Platelets are not only key components in coagulation but also reservoirs for various immunomodulatory molecules (e.g., TGF-β, platelet factor 4) and can interact directly with immune cells, affecting their recruitment, activation, and function (46–48). Some studies indicate that activated platelets can release immunosuppressive factors or inhibit effector T-cell infiltration and function through physical barriers and signal interference (49–51). Maintaining a high platelet count may reflect a poorer immune environment, leading to reduced efficacy of immunotherapy.

This study found that a higher post-treatment albumin level (ALB_post ≥39.65 g/L) is an independent protective factor for achieving MPR. Many studies indicate that albumin, as a key indicator reflecting nutritional status and systemic inflammation levels, suggests better physiological reserve and a more stable internal environment when sufficient. This provides the foundation for the immune system to function effectively (52, 53). Maintaining a higher albumin level during treatment may indicate good patient tolerance, milder systemic inflammatory response, and a microenvironment supportive of anti-tumor immune responses, favoring the action of immunotherapy.

Our study also has several important limitations that must be acknowledged. First, as a single-center retrospective analysis, although efforts were made to adjust for confounding factors, selection bias and unmeasured confounders cannot be completely avoided. Causality cannot be established, and conclusions require validation by multicenter prospective studies. Second, the treatment regimen in the study population was relatively homogeneous (predominantly SOX combined with sintilimab), so extrapolating results to other chemotherapy or immunotherapy regimens requires caution. The obtained cutoff values for blood indicators also need validation in independent cohorts. Third, the primary endpoint was pathologic response rather than long-term survival; the association of these markers with patient survival awaits confirmation through long-term follow-up. Furthermore, blood indicators are susceptible to interference from various factors such as infection and comorbidities; although strict exclusion criteria were applied, residual interference may still exist. Although the events-per-variable ratio (81 MPR events vs. 6 candidate variables) is acceptable, the potential for model overfitting cannot be fully excluded given the retrospective design and the exploratory nature of the analysis. Finally, this study only revealed statistical associations without elucidating mechanisms. For example, what functional subtype does a high neutrophil count correspond to? Does a high platelet count reflect a mere numerical recovery or a state of persistent activation? These questions require further exploration through translational research combining tumor microenvironment analysis and single-cell sequencing.

In summary, our study presents a novel finding: pre-treatment neutrophil level is a key indicator for predicting pathologic response to NICT in LAGC. Patients with low pre-treatment neutrophil levels achieved significantly higher pathologic response rates with 4 cycles of treatment compared to 2–3 cycles. Our study also confirmed that post-treatment platelet count and albumin level are independent predictors of pathologic response, and these associations are specific to immunochemotherapy.

## Data Availability

The raw data supporting the conclusions of this article will be made available by the authors, without undue reservation.
