# Supplementary material for: Hematological biomarkers for predicting pathologic response to neoadjuvant immunochemotherapy and cycle optimization in locally advanced gastric cancer
Source: Front Immunol. 2026 May 25;17:1795481. doi: 10.3389/fimmu.2026.1795481 (PMC13243286; doi:10.3389/fimmu.2026.1795481)
Supplement: Supplementary file 1 [file Table1.docx]

Supplementary Material

# Supplementary Data

# Supplementary Figures and Tables

## Supplementary Figures


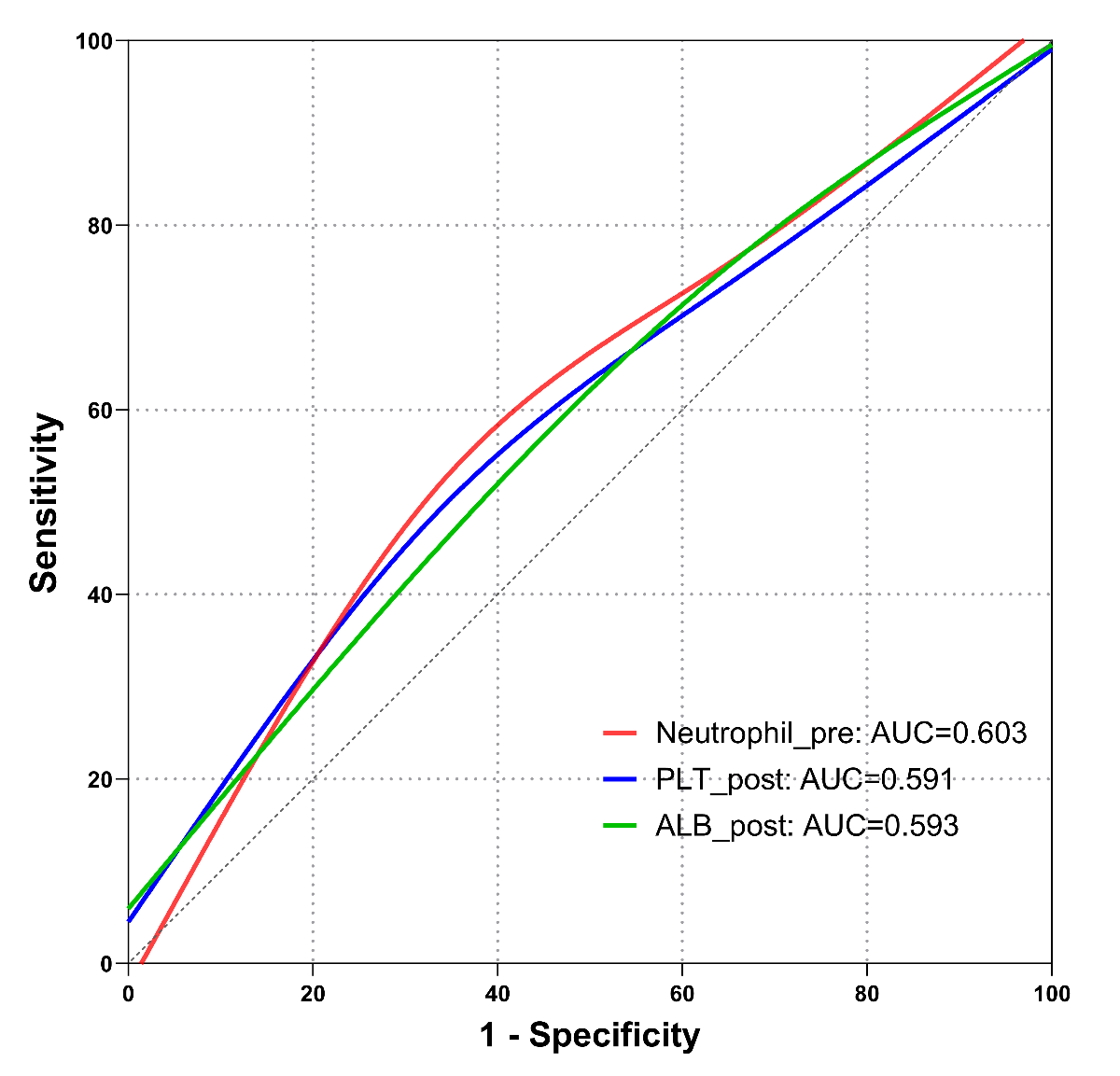


**Supplementary Figure 1. Receiver operating characteristic (ROC) curves of hematological biomarkers for predicting major pathologic response.** The curves depict the predictive performance of pre-treatment neutrophil count (Neutrophil_pre), post-treatment platelet count (PLT_post), and post-treatment albumin level (ALB_post) for MPR following neoadjuvant immunochemotherapy.

## Supplementary Tables

**Supplementary Table 1**

**Detailed composition of “other” chemotherapy regimens in the neoadjuvant immunochemotherapy (NICT) group**

| Regimen | Drugs | Number of patients and Percentage of total NICT cohort |
| --- | --- | --- |
| Paclitaxel + S-1 | Paclitaxel, S-1 (tegafur, gimeracil, oteracil) | 15 (6.1%) |
| Paclitaxel + Capecitabine | Paclitaxel, Capecitabine | 4 (1.6%) |
| Cisplatin + Etoposide | Cisplatin, Etoposide | 1 (0.4%) |
| Oxaliplatin + Docetaxel | Oxaliplatin, Docetaxel | 1 (0.4%) |
| Total |  | 21 (8.5%) |
